# Sex-specific upper airway morphological alterations are associated with obstructive sleep apnea severity in Down syndrome

**DOI:** 10.21203/rs.3.rs-10496930/v1

**Published:** 2026-08-06

**Authors:** Luis Miguel Echeverry-Quiceno, Sergi Llambrich, Sandra Giménez, Mateus Rozalem-Aranha, Yann Heuzé, Xavier Sevillano, Juan Fortea, Neus Martínez-Abadías

**Affiliations:** Ecologia i Ciències Ambientals (BEECA), Universitat de Barcelona (UB); Ecologia i Ciències Ambientals (BEECA), Universitat de Barcelona (UB); Hospital de la Sant Creu i Sant Pau. Institut de Recerca Sant Pau (IR SANT PAU); IR SANT PAU, Hospital de la Santa Creu i Sant Pau; Univ. Bordeaux, CNRS, Ministère de la Culture, PACEA; HER - Human-Environment Research Group, La Salle - Universitat Ramon Llull; IR SANT PAU, Hospital de la Santa Creu i Sant Pau; Ecologia i Ciències Ambientals (BEECA), Universitat de Barcelona (UB)

**Keywords:** Trisomy 21, Obstructive sleep apnea, Nasal cavity, Pharynx, Three-dimensional morphology, Geometric morphometrics

## Abstract

Individuals with Down syndrome (DS) have a high risk of obstructive sleep apnea (OSA), likely driven by craniofacial and upper airway abnormalities. However, three-dimensional upper airway morphology and its association with OSA severity remain poorly characterized. Here, we provide the first comprehensive three-dimensional characterization of upper airway volume and shape in adults with DS and euploid controls (EU). Three-dimensional upper airway models were reconstructed from T1-weighted MRI scans of 164 adults (63 EU, 101 DS). Upper airway, nasal cavity, and pharyngeal morphology were quantified using volumetry and geometric morphometrics (23 landmarks). OSA associations were assessed in a DS subsample (n = 91) using the apnea–hypopnea index and OSA severity categories. Adults with DS exhibited significantly reduced upper airway, nasal cavity, and pharyngeal volumes and distinct shape alterations relative to EU controls, with sex-specific patterns. In DS, increasing OSA severity was associated with reduced upper airway and nasal cavity volumes, particularly in males. OSA-related shape differences were confined to the nasal cavity of males with severe OSA, characterized by bilateral constriction and anteroposterior shortening. These findings identify upper airway morphology, particularly nasal cavity volume and shape, as imaging-derived anatomical biomarkers associated with OSA severity in adults with DS.

## Introduction

Sleep breathing disorders often encompass abnormalities in respiratory patterns or gas exchange during sleep, most notably apneas, hypopneas, and nocturnal hypoxemia [1]. Obstructive sleep apnea (OSA), the most common form in adults [2, 3], is defined by recurrent partial or complete collapse of the upper airway during sleep, leading to intermittent hypoxemia and sleep fragmentation, and is associated with significant neurocognitive, neurodegenerative, and cardiovascular consequences [4]. Craniofacial and upper airway structural abnormalities are well-established anatomical risk factors for OSA. Advanced imaging and cephalometric studies have demonstrated that skeletal features such as a retrognathic profile, reduced maxillary and mandibular dimensions, and an inferior–posterior hyoid position increase OSA risk [5, 6]. In addition, soft tissue and airway dysmorphologies, including lateral pharyngeal wall thickening, increased tongue volume, adenotonsillar hypertrophy, and nasal obstruction, further contribute to upper airway narrowing and collapse during sleep [7, 8, 9]. Down syndrome (DS; OMIM 190685) is a condition where all these risk factors converge.

This complex genetic disorder, caused by trisomy of chromosome 21, occurs in approximately one out of 700–1000 live births [10, 11], and is characterized by a broad spectrum of craniofacial anomalies and respiratory impairments [12]. The most typical orofacial features associated with DS are brachycephaly, flattened nasal bridge, mandibular and maxillary hypoplasia, muscular hypotonia, adenotonsillar hypertrophy, macroglossia, shortened palate, and narrow nasopharyngeal region [12, 13, 14, 15]. Collectively, these structural traits represent an increased risk for developing respiratory problems such as OSA. Indeed, adults with DS present a higher prevalence of sleep disturbances (74.5%) and OSA (78%) compared to the general population [16, 17].

OSA has been linked to cognitive deterioration through mechanisms including impaired sleep quality [18]. In addition, disrupted protein clearance may accelerate the accumulation of beta-amyloid (Aβ) [19] and tau proteins [20]. In turn, neurodegenerative conditions such as Alzheimer’s disease (AD) may further exacerbate the prevalence and severity of OSA in individuals with DS [21]. Consequently, the elevated burden of OSA, together with the accelerated cognitive decline characteristic of DS, highlights the importance of early diagnosis and management. However, diagnosing OSA in individuals with DS remains particularly challenging.

Overnight polysomnography (PSG) is the established gold standard for OSA detection [22]. However, this procedure can be tedious, uncomfortable, and has been questioned as a diagnostic tool in the DS population [14]. Performing PSG in individuals with DS presents additional challenges, since facial flatness, obesity, and restless behavior during testing may alter the PSG output, leading to misdiagnosis of OSA severity [21]. To overcome these limitations, particularly in populations at high risk of OSA, recent studies have explored non-invasive anatomical biomarkers for OSA diagnosis and risk stratification.

In non-syndromic populations, two-dimensional cephalometric analyses have shown promising results [23, 24], while three-dimensional facial morphology has emerged as an additional tool for OSA detection [25]. Ozdemir et al. [25] demonstrated significant localized facial differences, particularly within the nasal region, in individuals with OSA. In our previous work, we further showed that adults with DS and severe OSA exhibit distinctive three-dimensional craniofacial characteristics, particularly involving facial narrowing and mandibular retrusion [26].

Because craniofacial and upper airway development are closely integrated through shared developmental signaling pathways [27, 28], these craniofacial findings suggest that internal respiratory structures may also exhibit structural alterations associated with OSA susceptibility. However, craniofacial morphology provides only an external representation of the craniofacial complex and does not directly characterize upper airway anatomy.

Accordingly, several imaging studies have investigated upper airway morphology directly. Previous MRI and cephalometric studies have shown that soft-tissue and airway abnormalities, such as lateral pharyngeal wall thickening, increased tongue volume, adenotonsillar hypertrophy, and nasal obstruction, contribute to upper airway narrowing and collapse during sleep [7, 8]. In syndromic populations, Remy et al. [29] reported significant airway shape alterations in individuals with Crouzon and Apert syndromes, although no direct association with OSA could be demonstrated, likely because of the limited sample size. Whether upper airway morphology exhibits distinct volumetric and shape alterations associated with OSA in individuals with DS therefore remains unknown. To address this question, we explored the potential of upper airway morphology as a complementary anatomical biomarker for investigating respiratory disorders in DS.

Therefore, in this study we used a large sample of euploid controls (EU) and individuals with Down syndrome (DS) to conduct the first comprehensive three-dimensional volumetric and shape characterization of the upper airway in adults with DS and to investigate its association with OSA severity. Moreover, building on the sex-specific craniofacial differences associated with trisomy 21 reported by Echeverry-Quiceno et al. [26], we investigated whether analogous sex-specific patterns are also present in upper airway morphology and in its relationship with OSA in individuals with DS. Specifically, we aimed to (i) characterize volumetric and shape differences of the upper airway, nasal cavity, and pharynx between EU and DS individuals across adulthood, and (ii) investigate the association between upper airway volume and shape and OSA severity in individuals with DS. All analyses were performed separately in males and females to evaluate potential sex-specific patterns.

## Materials and Methods

### Sample composition

The sample comprised 164 adults, including 63 euploid individuals (EU) (19 males, 44 females) and 101 individuals with Down syndrome (DS) (62 males, 39 females) (Table 1). All individuals with DS were recruited from the population-based Down-Alzheimer Barcelona Neuroimaging Initiative (DABNI) cohort, whereas cognitively unimpaired EU participants were recruited from the Sant Pau Initiative on Neurodegeneration (SPIN) cohort. All participants were adults of European ancestry (Table 1) and had available T1-weighted head magnetic resonance imaging (MRI) scans suitable for upper airway reconstruction and analysis.

MRI data were acquired using either a 3T Philips Achieva scanner (Philips Healthcare) at Hospital del Mar (Barcelona) or a 3T Siemens Prisma scanner (Siemens Healthcare) at Hospital Clínic (Barcelona), with comparable acquisition parameters across sites (matrix size: 240 × 240; number of slices: 160; voxel size: 1 × 1 × 1 mm^3^; echo time [TE]: 3.7 ms; repetition time [TR]: 8.1 ms; inversion time [TI]: 240 ms; flip angle: 8°).

Participants with insufficient-quality MRI scans or inadequate three-dimensional upper airway reconstructions were excluded from the analyses to minimize methodological bias. All remaining eligible participants were included from the DABNI and SPIN cohorts. The EU and DS groups were therefore not individually matched by age or sex. To minimize potential confounding effects, age effects were accounted for in all volumetric and shape analyses, and sex-specific analyses were performed when appropriate.

The study was approved by the Sant Pau Ethics Committee following the standards for medical research in humans recommended by the Declaration of Helsinki. All participants or their legally authorized representatives gave written informed consent before enrollment.

### OSA diagnosis

Polysomnography data were available for a subsample of 91 individuals with DS (56 males, 35 females) and 44 EU individuals (13 males, 31 females). OSA presence and severity were assessed using the apnea–hypopnea index (AHI) obtained from overnight polysomnography tests. However, because only a limited number of EU participants presented OSA (11 mild and 3 severe cases), analyses of OSA-related anatomical variation were restricted to the DS sample to ensure adequate statistical power and balanced group comparisons. These sleep studies were conducted in temperature-controlled and sound-attenuated rooms at the Sant Pau Sleep Unit, following the protocols described in Giménez et al. [17]. The AHI represents the total number of apneas—defined as a ≥ 90% airflow reduction for more than 10 seconds—and hypopneas—characterized by ≥ 30% airflow decrease for at least 10 seconds, accompanied by either a ≥ 3% drop in oxygen saturation or an arousal—per hour of sleep.

Participants with DS were initially classified according to the presence or absence of OSA based on their AHI, using a cutoff of 5 events/hour (no OSA: AHI < 5; OSA: AHI ≥ 5) (Table 1). For a more detailed classification, participants were further categorized into three groups based on OSA severity: no OSA (AHI < 5), mild OSA (AHI 5–29.9), and severe OSA (AHI ≥ 30) (Table 1). Following Echeverry-Quiceno et al. [26], and to increase within-group sample sizes and statistical power, the “mild OSA” category combined the conventional mild (AHI 5–15) and moderate (AHI 15–29.9) OSA classifications. Given the limited sample size, maintaining separate mild and moderate categories would have reduced statistical power and increased group imbalance. The merged category therefore facilitated more robust comparisons across OSA severity levels.

Given the previously reported association between BMI and OSA in DS [16, 17], we evaluated whether BMI influenced upper airway, nasal cavity, and pharyngeal volume and shape in a subsample of DS participants with available BMI data (n = 87) (Supplementary Table S1). A permutation-based analysis of variance (ANOVA) was used for these analyses.

### Upper airway 3D models, landmarking and volumetric data

Three-dimensional reconstructions of the upper airway were manually generated from T1-weighted MRI. The gray-scale threshold was adjusted for each individual to restrict the segmentation to aerated regions, and respiratory structures were manually segmented slice by slice using Amira 5.2.1 (v6.3, Visualization Sciences Group, FEI), using the second intervertebral disc as the inferior boundary for segmentation (Supplementary Fig. S1).

**Figure 1:**
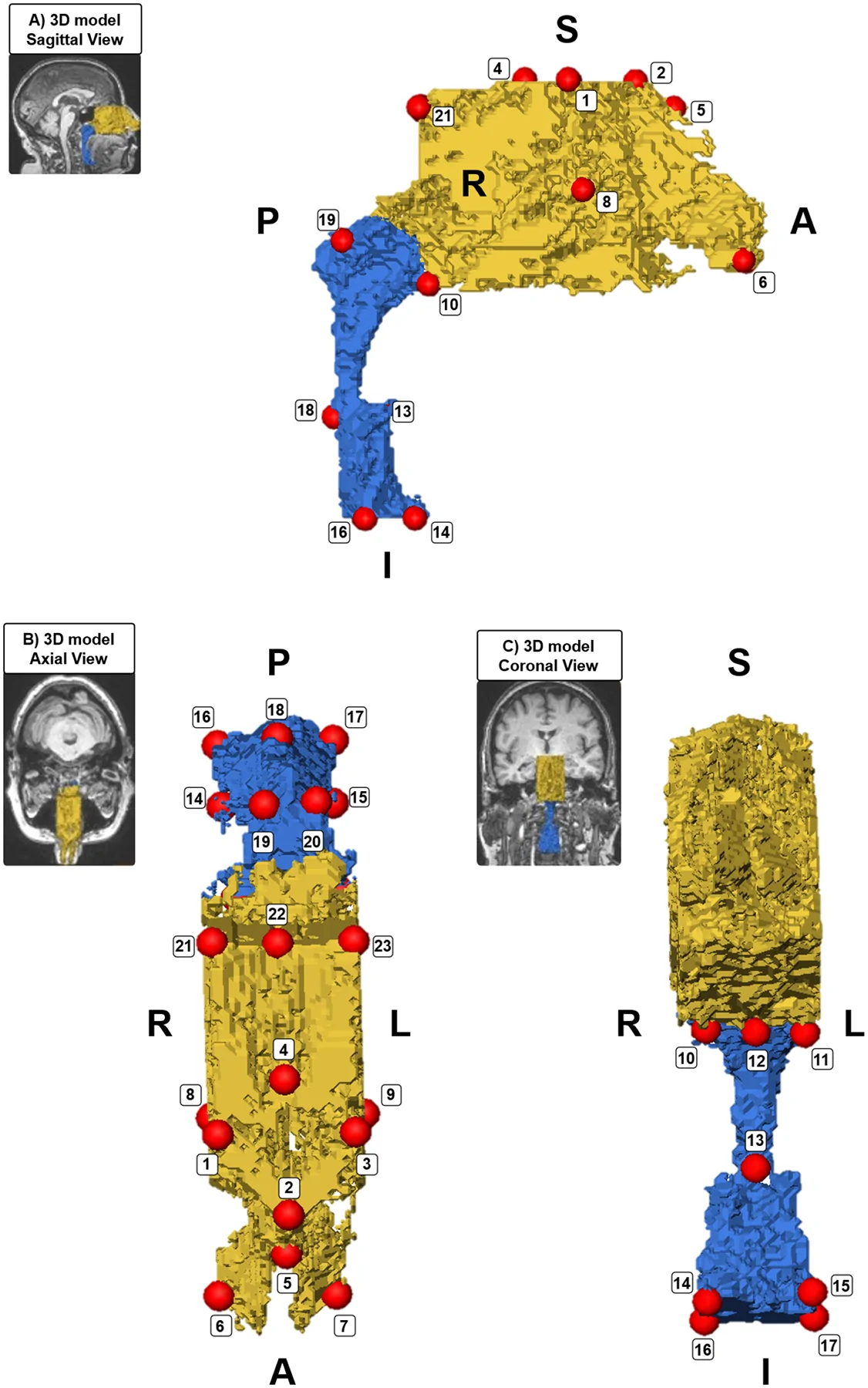
Anatomical landmarks used for three-dimensional upper airway analyses. Positions of the 23 anatomical landmarks used for geometric morphometric and univariate/multivariate statistical analyses shown in A) sagittal, B) axial and C) coronal views. See Supplementary Table S2 for landmark definitions.

Volumetric data (mm^3^) were extracted from the segmentations, and 3D models of the upper airway were reconstructed. To capture the upper airway morphology, we recorded the 3D coordinates of 23 anatomical landmarks placed on the 3D reconstructions (Fig. 1; Supplementary Table S2). To further localize morphological and volumetric differences among individuals, the upper airway segmentation was divided into two anatomical substructures: a) the nasal cavity, with the nares (nostrils) as the anterior boundary, and the choanae as the posterior boundary; and b) the pharynx, with the choanae as the superior boundary, and the second intervertebral disc serving as the inferior boundary.

### Geometric Morphometrics and statistical analyses

#### Volumetric analyses

To assess volume variation in the entire upper airway, as well as in the different substructures (the nasal cavity and pharynx) explained by genetic diagnostic group (EU or DS), OSA diagnostic group, sex, age (ontogeny), and the apnea–hypopnea index (AHI), we conducted a permutation-based analysis of variance (ANOVA) using the Randomized Residual Permutation Procedure (RRPP; 10,000 permutations) [30], as implemented with the *lm.rrpp* function from the RRPP R package (version 2.0.3). In addition, linear regressions were performed to evaluate, both statistically and visually, the correlation between upper airway/substructure volumes and sex, age, and AHI.

To test for statistically significant differences in respiratory substructure volumes across groups, we first applied the Shapiro–Wilk test for normality and Levene’s test for homogeneity of variances, to select the appropriate parametric or non-parametric approach. For comparisons between two groups, we conducted Student’s *t*-tests for parametric analyses and Wilcoxon signed-rank tests for non-parametric cases using the functions *t.test* and *wilcox.test* from the stats R package (version 4.4.1). For comparisons involving more than two groups, we used one-way ANOVA (parametric) or the Kruskal–Wallis rank-sum test (non-parametric), implemented with the functions *aov*, and *kruskal.test* from the stats R package (version 4.4.1). Post hoc comparisons were performed using Tukey’s Honest Significant Difference (HSD) test for parametric cases and pairwise Wilcoxon rank-sum tests for non-parametric cases, to identify specific group differences.

To visualize volumetric differences of the upper airway and its substructures across diagnostic groups: a) EU/DS, b) No OSA/OSA and c) No OSA/mild OSA/severe OSA; we generated boxplots using the function from the *ggplot2* R package (version 3.5.1).

#### Shape analyses

For shape analyses, we applied Geometric morphometrics (GM), a robust set of statistical tools designed for measuring and comparing 3D shapes with high precision and efficiency [31, 32, 33, 34]. A Generalized Procrustes Analysis (GPA) [35] was first performed to superimpose all landmark configurations into a common morphospace by scaling, rotating, and translating the landmark coordinates [36]. Then, to quantify the shape variation of the upper airway and its substructures attributable to variables such as genetic diagnostic group (EU or DS), OSA diagnostic group, sex, age and AHI, we performed a permutation-based ANOVA test using the RRPP algorithm (10,000 permutations) [30], as implemented with the *ProcD.lm* function from the Geomorph R package (version 4.0.8). Additionally, linear regressions were performed to evaluate, both statistically and visually, the correlation between upper airway/substructure shape and the variables listed above.

Volumetric and shape analyses were performed both on combined data and separately for EU and DS samples, when applicable. Because age differed between diagnostic groups and is a known source of upper airway variation, age effects were first removed using linear regression models and age-adjusted residuals were subsequently used for all group comparisons and statistical analyses. Analyses were further stratified by sex when significant sex effects were detected (Supplementary Table S3), and comparisons were subsequently performed separately for males and females when appropriate. In addition, each upper airway substructure was analyzed independently to assess localized patterns.

Regarding OSA analyses, we only included the DS group and conducted a first analysis using two categories: A) No OSA and B) OSA. Subsequently, a three-group classification was implemented: A) no OSA, B) mild OSA, and C) severe OSA. Post hoc pairwise comparisons were performed to identify significant differences in volume and shape between groups.

To visualize shape differences between diagnostic groups, we conducted Canonical Variate Analysis (CVA), a statistical discriminant method that maximizes the separation among predefined groups [37]. Upper airway morphings associated with the negative and positive extremes of the first two CVs were created with IDAV Landmark Editor version and MorphoJ version 1.07a b [38]. In addition, surface-distance heatmaps were generated in Amira 5.2.1 (v6.3, Visualization Sciences Group, FEI) to visualize the magnitude and spatial distribution of shape differences between groups. For each vertex of one surface, the distance to the closest point on the comparison surface was computed using the *SurfaceDistance* module. Warmer colors indicate greater surface displacement between surfaces, whereas cooler colors indicate smaller displacement.

## Results

### Trisomy 21 is associated with sex-specific volumetric reductions and dysmorphologies of the upper airway

We first characterized the volumetric and morphological differences in the upper airway, nasal cavity, and pharynx between EU controls and individuals with DS.

**Figure 2:**
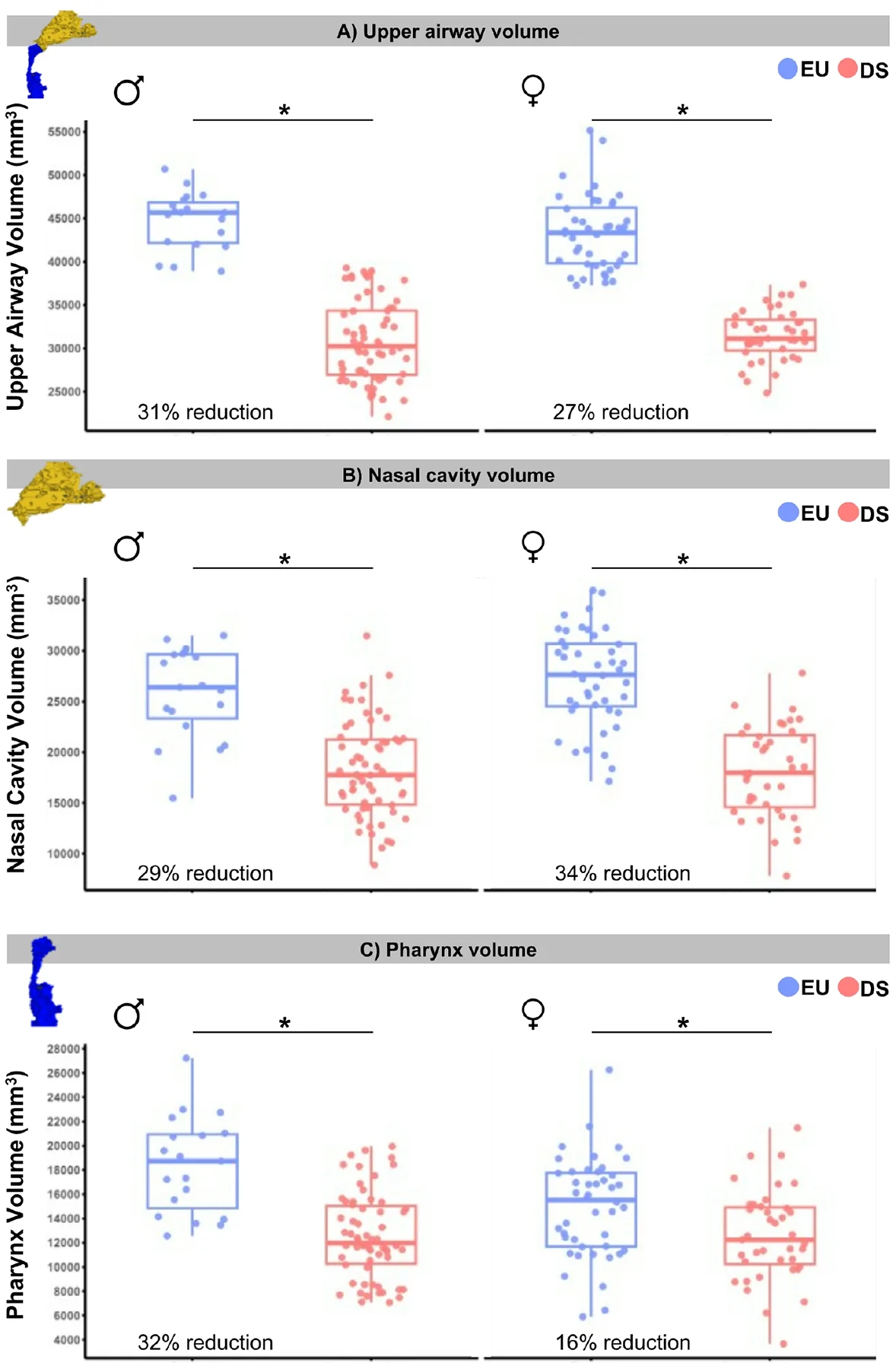
Sex-specific volumetric differences in the upper airway between EU controls and individuals with DS. Volumetric comparison of the A) entire upper airway, B) nasal cavity and C) pharynx, shown separately for males and females. *Statistically significant difference between groups (p < *0.0001)*.

Both male and female individuals with DS exhibited significantly reduced volumes compared to EU controls in the entire upper airway (*p* < 0.0001; Fig. 2A), the nasal cavity (*p* < 0.0001; Fig. 2B) and the pharynx (*p* < 0.0001; Fig. 2C). Substructure analysis revealed sex-specific patterns of volumetric reduction. In comparison to EU controls, males with DS showed a greater reduction of the entire upper airway (31%) compared to females with DS (27%) (Fig. 2A). In the nasal cavity, females showed a greater reduction (34%) than males (29%) (Fig. 2B). In contrast, in the pharyngeal region, females with DS exhibited only a 16% reduction, whereas males showed a reduction of 32% (Fig. 2C).

**Figure 3:**
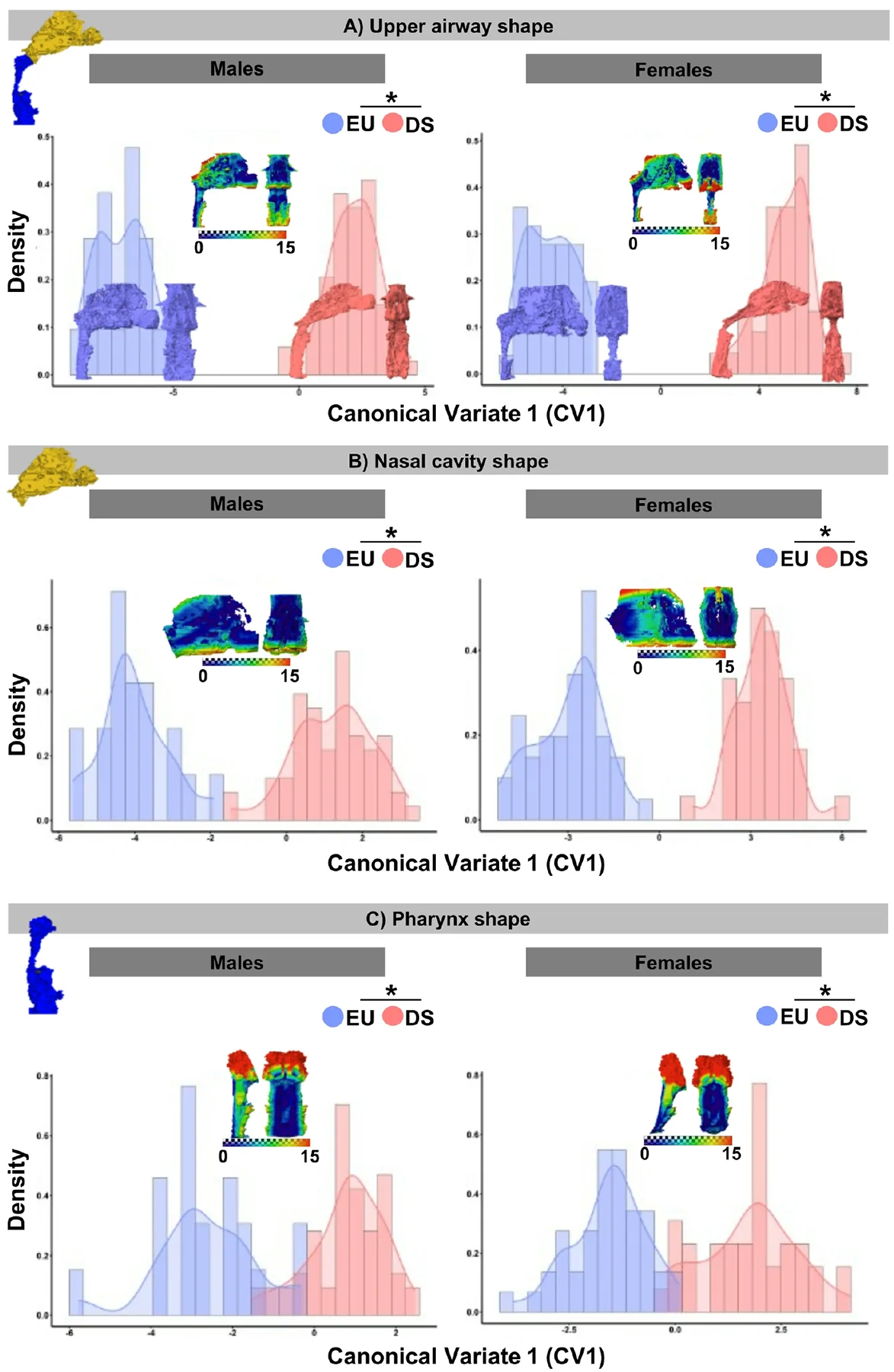
Sex-specific shape differences in the upper airway between EU controls and individuals with DS. Canonical variate analyses (CVA) performed separately for males and females for A) the entire upper airway, B) the nasal cavity and C) the pharynx. The x-axis represents Canonical Variate 1 (CV1), accounting for 100% of the shape variation, and the y-axis shows the density distribution for each group. For the entire upper airway (A), morphings at the negative extreme of CV1 (frontal and lateral views of EU individuals, shown in blue) are contrasted with morphings at the positive extreme of CV1 (individuals with DS, shown in red). Upper airway morphings are magnified by a factor of 2. CVA results are accompanied by surface-distance heatmaps illustrating the magnitude and spatial distribution of shape differences for A) the upper airway, B) the nasal cavity and C) the pharynx. Warmer colors indicate greater surface displacement between the mean EU and DS shapes, whereas cooler colors indicate smaller displacement. *Statistically significant difference between groups (p < *0.0001)*.

Furthermore, Procrustes ANOVA based on shape data revealed that the volumetric reduction is accompanied by significant morphological differences in individuals with DS compared to EU controls, which affect the entire upper airway (*Z* = 5.4286, *p* < 0.0001; Fig. 3A), the nasal cavity (*Z* = 4.3986, *p* < 0.0001; Fig. 3B), and the pharynx (*Z* = 4.8609, *p* < 0.0001; Fig. 3C) in both males and females. Sex-specific differences in the severity of dysmorphologies were observed across respiratory substructure in individuals with DS, with females exhibiting more pronounced alterations (Fig. 3A,B,C). In the nasal cavity, both males and females with DS showed posterior flattening at the choanal level, accompanied by an antero-inferior reduction in the nostril region (Fig. 3A,B). In addition, an upward angulation and bilateral constriction of the nasal cavity were detected in both sexes (Fig. 3A,B). Regarding the pharyngeal section, both sexes exhibited a posterior bending with a bilateral shrinking (Fig. 3A,C).

Then, we explored whether these volumetric and morphological patterns changed throughout ontogeny in both EU and DS populations. Permutation-based ANOVA revealed no significant volumetric or morphological airway changes across age but remained stable in all groups except in females with DS (Supplementary Figs. S2, S3; Supplementary Tables S4, S5). Females with DS showed a decrease in the volume of the entire upper airway and nasal cavity, accompanied by an increase in pharyngeal volume over time (Supplementary Fig. S3). These ontogenetic changes affected volume but not shape in this group.

### Obstructive sleep apnea severity is associated with further volume airway reductions in males with Down syndrome

Regarding OSA in DS, we first examined whether the shape and volume variation of the upper airway, nasal cavity, and pharynx was dependent on BMI. Permutation-based ANOVA showed that BMI had no significant effect on either volumetric or morphological variation of these substructures (Supplementary Table S3); therefore, subsequent analyses were performed without adjusting for BMI.

Then, we evaluated the association between upper airway morphology and AHI, a quantitative clinical measure of OSA severity. In our cohort, males presented higher AHI scores than females (males: $\overset{-}{\text{x}}=29.5$; females: $\overset{-}{\text{x}}=18.1$), and this difference was statistically significant (*p* = 0.0010), supporting further sex-stratified analyses. Permutation-based ANOVA revealed that AHI explained a significant proportion of the entire upper airway volume variance, with marked sex differences: 56% in males and 16% in females (Supplementary Figs. S4, S5; Supplementary Table S6). Similarly, AHI explained a significant proportion of nasal cavity volume variance, again with sex-specific effects (31% in males and 16% in females), whereas no significant association was observed for pharyngeal volume (Supplementary Table S6). In contrast, AHI analyzed as a continuous variable did not significantly explain shape variance in any upper airway substructure (Supplementary Table S6). To further explore whether non-linear or threshold-based effects might underlie this pattern, analyses were repeated using a categorical OSA classification, comparing individuals with (AHI ≥ 5) and without OSA (AHI < 5).

**Figure 4:**
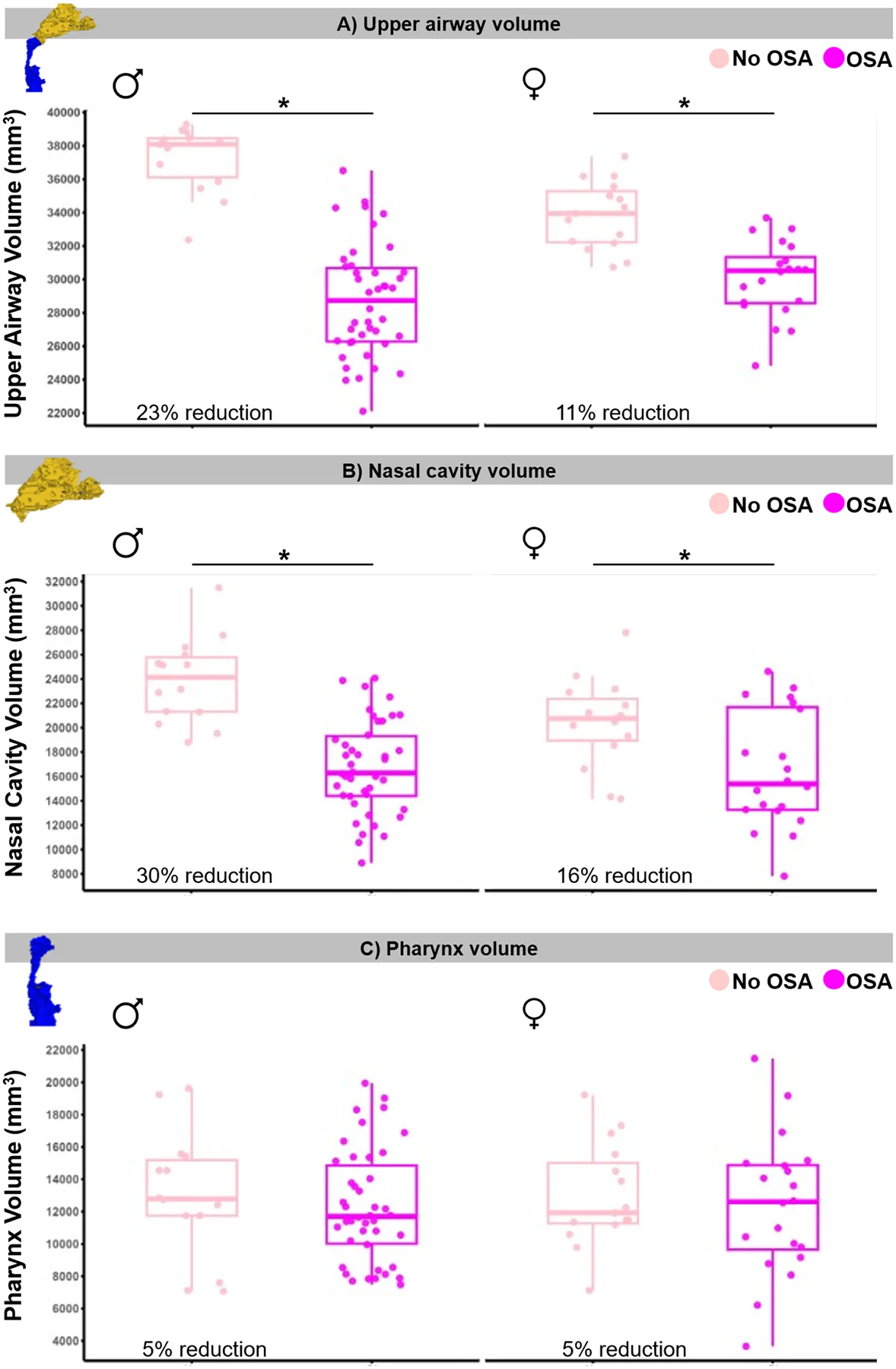
Sex-specific volumetric differences in the upper airway between individuals with DS with and without OSA. Volumetric comparison of the A) entire upper airway, B) nasal cavity and C) pharynx, shown separately for males and females. *Statistically significant difference between groups (p < *0.0001)*.

Both males and females diagnosed with OSA showed a significant volume reduction of the entire upper airway (*p* < 0.0001; Fig. 4A) and nasal cavity (*p* < 0.0001; Fig. 4B), but not of the pharynx (*p* = 0.5495 and *p* = 0.6331 respectively; Fig. 4C). Males diagnosed with OSA showed nearly twice the volumetric reduction in the entire upper airway (23%) and the nasal cavity (30%) compared with females with OSA, who exhibited reductions of 11% and 16%, respectively.

**Figure 5:**
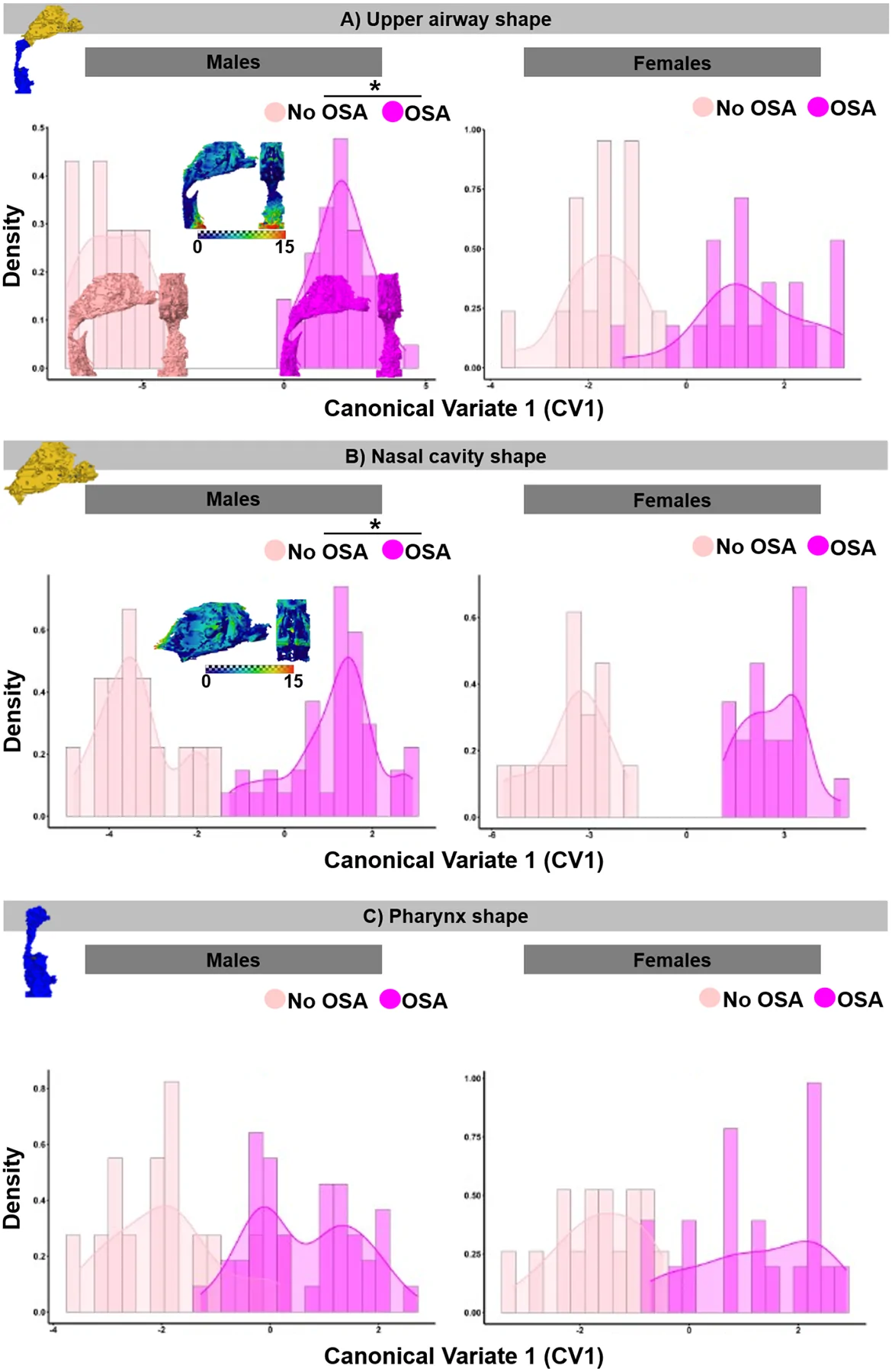
Sex-specific shape differences in the upper airway between individuals with DS with and without OSA. Canonical variate analyses (CVA) performed separately for males and females for A) the entire upper airway, B) the nasal cavity and C) the pharynx. The x-axis represents Canonical Variate 1 (CV1), accounting for 100% of the shape variation, and the y-axis shows the density distribution for each group. For the entire upper airway (A), morphings at the negative extreme of CV1 (frontal and lateral views of individuals with DS and no OSA, shown in pink) are contrasted with morphings at the positive extreme of CV1 (individuals with DS and OSA, shown in magenta). Upper airway morphings are magnified by a factor of 2. CVA results are accompanied by surface-distance heatmaps illustrating the magnitude and spatial distribution of shape differences for A) the upper airway, B) the nasal cavity and C) the pharynx. Warmer colors indicate greater surface displacement between the mean shapes of individuals with DS with and without OSA, whereas cooler colors indicate smaller displacement. *Statistically significant difference between groups (p < *0.0001)*.

Regarding shape, significant morphological differences between DS individuals with and without OSA were only detected in males, affecting the entire upper airway (*Z* = 2.1644, *p* = 0.0157; Fig. 5A) and the nasal cavity (*Z* = 2.0949, *p* = 0.0159; Fig. 5B), but not the pharynx (*Z* = 1.9906, *p* = 0.4493; Fig. 5C). Males with DS and OSA exhibited a slight constriction across the nasal cavity compared with those without OSA (Fig. 5A,B), with a more pronounced anteroposterior reduction of this substructure (Fig. 5A,B).

**Figure 6:**
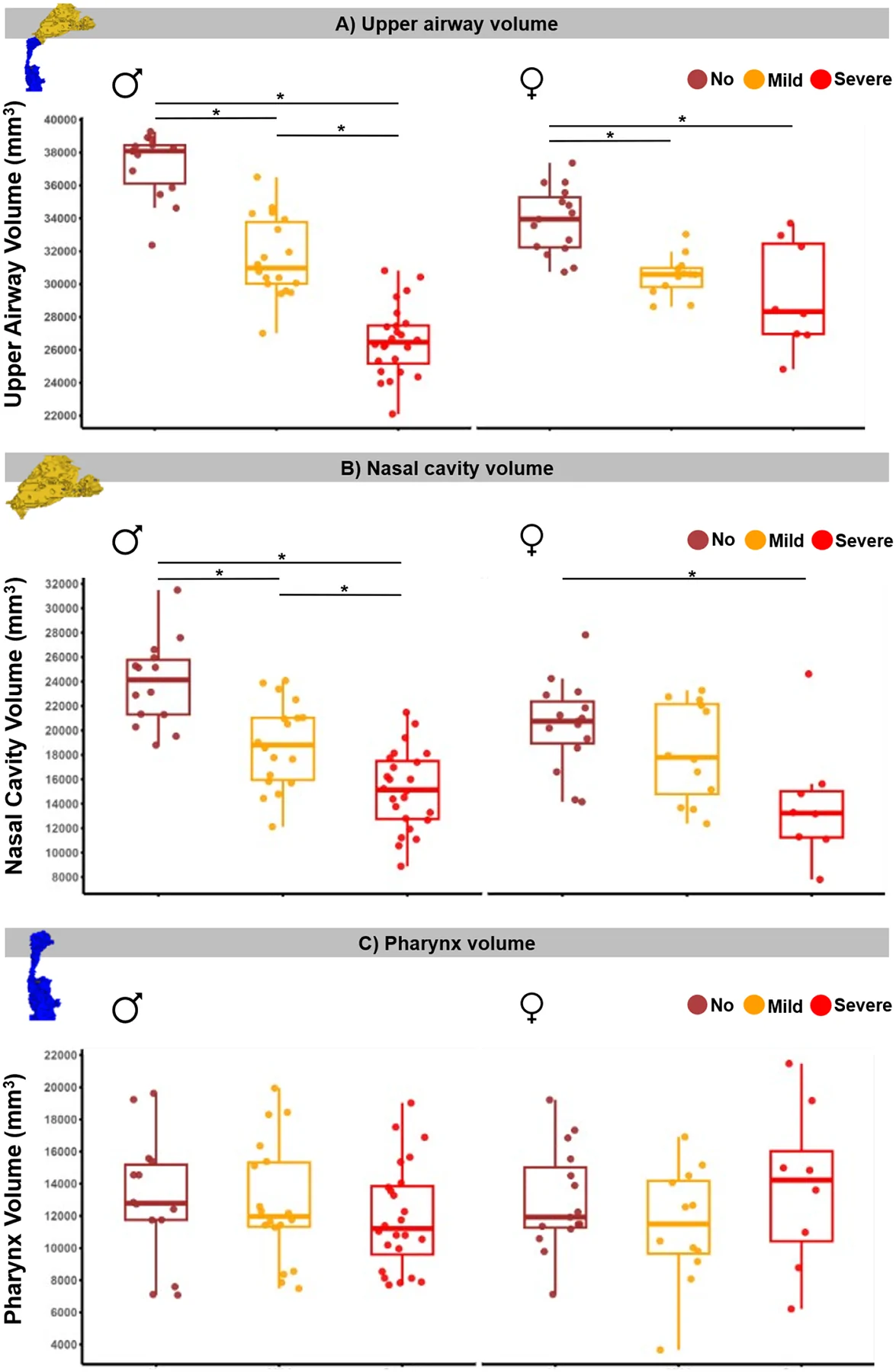
Sex-specific volumetric differences in the upper airway across OSA severity in individuals with DS. Volumetric comparison of the A) entire upper airway, B) nasal cavity and C) pharynx among individuals with no OSA, mild OSA and severe OSA, shown separately for males and females. *Statistically significant difference between groups (p < *0.0001)*.

To further explore in detail these volumetric and morphological differences associated with OSA, we evaluated individuals with DS based on the three-group OSA classification separately for males and females. Our results revealed a progressive reduction in volume across OSA severity levels in both sexes for the entire upper airway (*p* < 0.0001; Fig. 6A) and the nasal cavity (*p* < 0.0001; Fig. 6B), but not for the pharynx (M: *p* = 0.5980; F: *p* = 0.3802; Fig. 6C). In males with DS, significant volumetric differences in the entire upper airway were detected for all pairwise comparisons across OSA severity (*p* < 0.0001; Fig. 6A). In contrast, in females with DS, no significant difference was found between the mild and severe OSA groups (*p* = 0.31; Fig. 6A). For the nasal cavity, males similarly showed significant volumetric differences in all pairwise comparisons (*p* < 0.0001; Fig. 6B). Conversely, in females, significant differences were observed only between individuals with no OSA and those with severe OSA (*p* < 0.0001; Fig. 6B).

**Figure 7:**
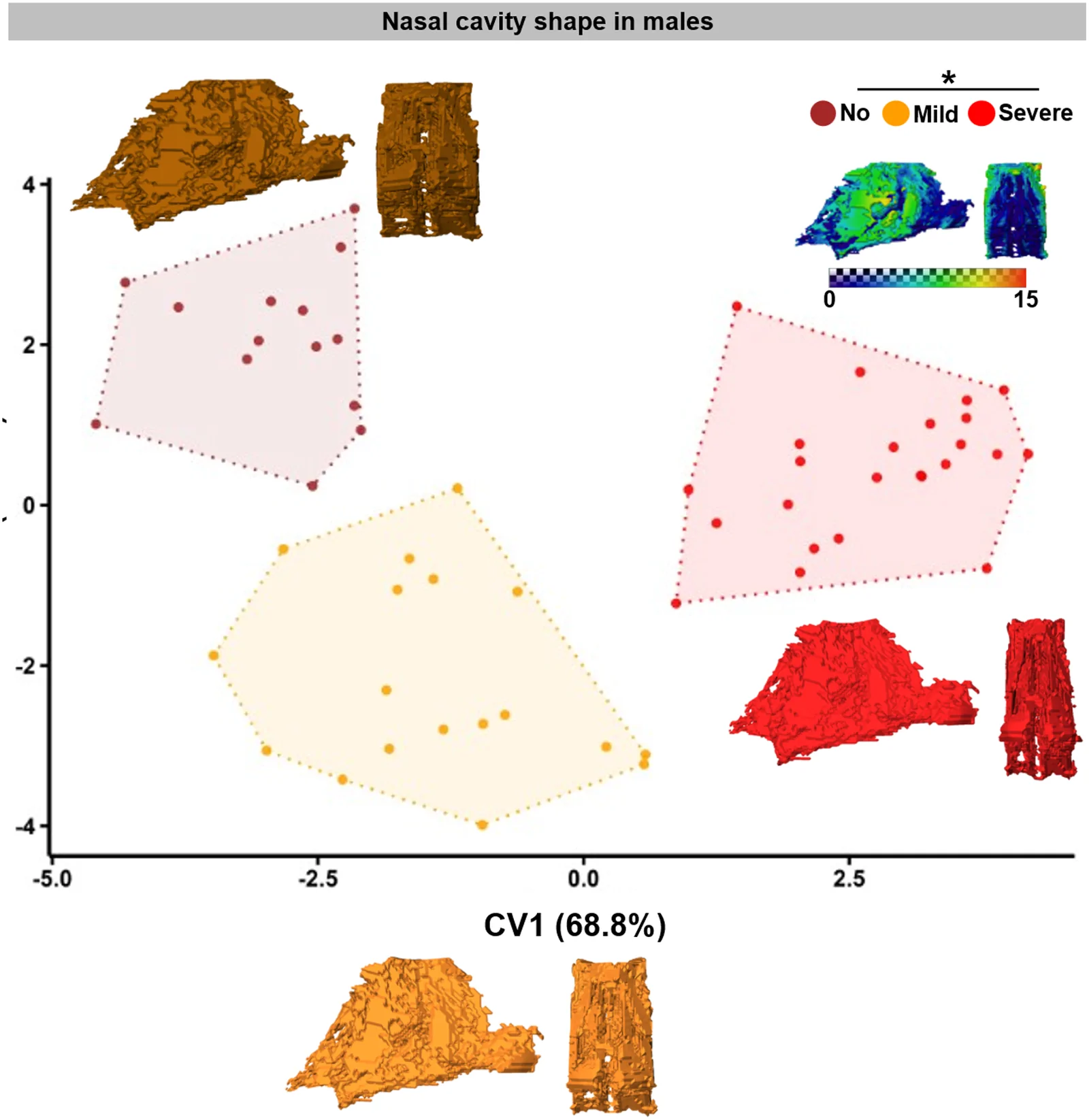
Nasal cavity shape differences across OSA severity in males with DS. Canonical variate analysis (CVA) comparing males with DS without OSA and with severe OSA. CVA results are accompanied by morphings associated with the negative extreme of CV1 (individuals with DS and no OSA) and the positive extreme of CV1 (individuals with DS and severe OSA). Surface-distance heatmaps illustrate the magnitude and spatial distribution of shape differences between the negative (no OSA) and positive (severe OSA) extremes of CV1. Warmer colors indicate greater surface displacement, whereas cooler colors indicate smaller displacement. *Statistically significant difference between groups (p < *0.0001)*.

Regarding shape, significant morphological differences were detected only in males for the nasal cavity, specifically between individuals with DS without OSA and those with severe OSA (*Z* = 2.6937, *p* = 0.0425; Fig. 7). Males with DS and severe OSA exhibited a bilateral constriction and a slight anteroposterior reduction of the nasal cavity, particularly in the choanal region (Fig. 7).

## Discussion

This study presents the first comprehensive three-dimensional quantitative characterization of the upper airway in adults with Down syndrome (DS), integrating both volumetric and shape analyses to investigate its association with obstructive sleep apnea (OSA). Our findings demonstrate that the upper airway, nasal cavity, and pharynx are significantly reduced in volume and altered in shape in individuals with DS compared to euploid controls (EU), with sex-specific differences. Specifically, males with DS showed more pronounced volumetric reductions in the pharyngeal region, whereas females exhibited greater nasal cavity volume reduction accompanied by more marked shape alterations across the upper airway and substructures.

Within the DS cohort, OSA severity was primarily associated with volumetric reductions of the upper airway, particularly in the nasal cavity and more prominently in males. Although upper airway shape was not significantly associated with AHI as a continuous variable, localized shape differences emerged when individuals were stratified into categorical OSA severity groups, indicating an association between upper airway morphology and categorical OSA severity. Collectively, these findings reveal distinct structural vulnerabilities with sex-specific patterns of the upper airway in DS and provide quantitative evidence supporting airway metrics as potential anatomical biomarkers for OSA risk detection in this population.

The marked reductions in upper airway, nasal cavity, and pharyngeal volume observed in individuals with DS were accompanied by distinct morphological alterations compared with EU controls. These dysmorphologies included posterior flattening and bilateral constriction of the nasal cavity, antero-inferior narrowing at the nostrils, and posterior angulation of the pharyngeal structures. Because craniofacial and upper airway structures develop as an integrated anatomical system regulated by shared developmental signaling pathways [27, 28], these upper airway alterations may reflect the characteristic craniofacial phenotype of DS, which includes brachycephaly, a flattened nasal bridge, midfacial hypoplasia, muscular hypotonia, adenotonsillar hypertrophy, macroglossia and a shortened palate [12, 13, 14, 15].

Although previous imaging studies have examined upper airway anatomy in individuals with DS, these investigations have primarily focused on pediatric populations using MRI [39] or cone-beam CT [40, 41]. Moreover, they have largely emphasized airway dimensions or volume rather than comprehensive three-dimensional shape characterization. To our knowledge, our study provides the first high-resolution three-dimensional quantitative characterization of upper airway volume and shape in adults with DS, including both nasal and pharyngeal components. Our approach also enabled a detailed characterization of the nasal cavity, an upper airway substructure that has received comparatively less attention in previous studies of adults with DS. Although pharyngeal segmentation was limited by MRI coverage, which may have reduced sensitivity for detecting subtle pharyngeal differences, the nasal cavity was fully captured in all participants, allowing a robust quantitative characterization of this anatomical region.

Furthermore, our results demonstrated sex-specific differential effects on upper airway morphology operating at two levels: (i) a genotypic effect associated with trisomy 21, which was more pronounced in females, and (ii) an OSA severity–related effect, which predominantly affected males.

The greater upper airway dysmorphology observed in females with DS is consistent with previous reports of more pronounced craniofacial alterations in females with trisomy 21 [26]. Additionally, previous studies have shown that females with DS tend to exhibit lower rates of bone mineral accrual [42], higher fat mass, and lower lean mass than males [43], supporting the concept of sex-specific anatomical vulnerability. Together, the combination of more pronounced craniofacial dysmorphology and increased adiposity in females may contribute to a more constricted and dysmorphic upper airway associated with trisomy 21.

Conversely, the association between upper airway morphology and OSA severity was more pronounced in males. Consistent with our findings, males also exhibited higher AHI values than females (Males: $\overset{-}{\text{x}}=29.5$; Females: $\overset{-}{\text{x}}=18.1$; p = 0.00104), and OSA severity explained a greater proportion of upper airway volumetric variation, primarily driven by reductions in nasal cavity volume. Although AHI was not associated with upper airway shape as a continuous variable, localized nasal cavity shape alterations were observed in males with severe OSA, suggesting that anatomical differences are more readily detected across clinically defined severity categories than along a continuous AHI gradient.

This male predominance is consistent with the well-established epidemiological pattern observed in the general population, where both OSA prevalence and severity are higher in males than in females [2, 44, 45]. Previous studies have also shown that males generally exhibit greater upper airway soft-tissue volumes, narrower nasal and choanal cross-sectional areas, and reduced neuromuscular and hormonal protection against upper airway collapse [7, 45, 46, 47, 48]. Within the context of DS, where craniofacial hypoplasia and generalized muscular hypotonia are already present [12, 13, 14, 15], these anatomical differences may further increase susceptibility to OSA-related upper airway compromise.

In addition, physiological mechanisms may also contribute to this sex-specific pattern. Females have been reported to exhibit a lower arousal threshold than males, leading to earlier termination of obstructive events, shorter respiratory disturbances, and less severe oxygen desaturation, despite potentially greater sleep fragmentation [45, 49]. Together, these anatomical and physiological differences provide a plausible explanation for the stronger association between upper airway morphology and OSA severity observed in males with DS.

Beyond the observed sex-specific differences, one of the main findings of this study was that the most consistent anatomical associations with OSA severity were identified within the nasal cavity. Individuals with DS and severe OSA exhibited greater reductions in nasal cavity volume and localized nasal shape alterations, whereas we did not detect significant associations between pharyngeal morphology and OSA severity in our dataset. Because pharyngeal segmentation was partially limited by MRI coverage, the absence of significant associations involving the pharynx should be interpreted cautiously.

While the strongest anatomical associations identified in our study involved the nasal cavity, previous studies in adults with OSA have shown that nasal resistance explains only a modest proportion of disease severity [50]. Because we did not directly assess nasal resistance, airway collapsibility, or other physiological determinants of upper airway obstruction, our findings should be interpreted as identifying structural correlates of OSA severity rather than evidence that nasal cavity morphology is a primary determinant of disease severity. Instead, our findings detect nasal cavity morphology as one of the anatomical features most consistently associated with OSA severity in adults with DS, while recognizing that OSA remains a multifactorial disorder influenced by additional anatomical and physiological mechanisms.

The anatomical pattern identified in our study also differs from that typically reported in non-syndromic OSA, where pharyngeal collapsibility, parapharyngeal soft tissues, and obesity-related mechanisms are generally considered the predominant anatomical contributors [7, 22]. Likewise, our findings differ from those reported in children with DS. Bokov et al. [51] observed that pharyngeal volume, rather than nasal obstruction, was more strongly associated with OSA in pediatric patients with DS. These apparent discrepancies may reflect both methodological differences between studies and age-related changes in craniofacial growth and upper airway anatomy. Whereas Bokov et al. [51] focused primarily on pharyngeal volume in children, the present study provides a comprehensive three-dimensional characterization of upper airway volume and shape in adults with DS.

Moreover, recent geometric morphometric analyses in craniosynostosis syndromes similarly demonstrate that the anatomical regions most strongly associated with airway compromise differ across genetic conditions, with Crouzon syndrome predominantly affecting the oropharynx and Apert syndrome the nasopharynx [29]. Collectively, these findings suggest that the anatomical correlates of OSA are likely to vary according to developmental stage and underlying syndrome, while methodological differences between studies may also contribute to the observed discrepancies.

Taken together, our findings support the potential value of three-dimensional upper airway phenotyping as a complementary imaging approach for improving the anatomical characterization and risk stratification of OSA in adults with DS. Rather than indicating that nasal cavity morphology alone determines OSA severity, our results suggest that nasal cavity volume and shape represent measurable anatomical features associated with disease severity within the broader craniofacial phenotype of DS. Given the challenges of performing polysomnography in this population, three-dimensional airway imaging may provide complementary information for identifying individuals at increased risk of OSA and contribute to more personalized clinical assessment. Considering the well-established association between OSA, cognitive decline, and the accelerated development of Alzheimer's disease in individuals with DS, improving the early identification of those at increased risk of OSA may have important implications beyond sleep medicine, potentially supporting strategies aimed at preserving cognitive function and delaying neurodegenerative progression.

### Limitations and future directions

The main strengths of this study include comprehensive three-dimensional airway phenotyping, separate evaluation of nasal and pharyngeal components, sex-stratified analyses, and integration of OSA severity, enabling a detailed anatomical characterization of upper airway variation in DS. Nevertheless, several limitations should be acknowledged. The cross-sectional design precludes longitudinal inference regarding airway development, OSA onset, and progression across the lifespan. Information on several factors known to influence OSA severity, including medication use, lifestyle factors, comorbidities, and detailed anthropometric measures, was unavailable for all participants and therefore could not be incorporated into the analyses. Accordingly, our findings should be interpreted as anatomical correlates of OSA severity rather than a comprehensive characterization of all factors contributing to OSA pathophysiology in DS.

The EU and DS groups were derived from independent cohorts and were not individually matched for age or sex. Although statistical models accounted for these variables, potential effects of differences in sample composition cannot be completely excluded. In addition, although polysomnography data were available for a subset of EU participants, the limited number with OSA precluded robust comparisons between syndromic and non-syndromic OSA phenotypes. Finally, airway reconstruction relied on manual segmentation, a time-consuming and operator-dependent process that limits scalability and clinical translation. Future work should incorporate automated segmentation and landmarking approaches, such as recent deep learning workflows [52].

Future studies should validate these findings in larger longitudinal cohorts integrating three-dimensional anatomical imaging with functional respiratory measurements, relevant clinical covariates, and sleep-phase imaging. Such multimodal approaches may clarify causal pathways, improve understanding of sex-specific mechanisms, and support the development of imaging-based biomarkers for early, non-invasive OSA detection and personalized intervention strategies in DS.

## Conclusions

Individuals with DS exhibit significant volumetric and morphological alterations of the upper airway compared with euploid controls, with distinct sex-specific patterns. In adults with DS, OSA severity was primarily associated with upper airway anatomical variation, particularly reduced nasal cavity volume and localized nasal shape alterations in males. Overall, these findings provide the first comprehensive three-dimensional characterization of upper airway morphology in adults with DS and support the potential value of imaging-derived upper airway phenotypes as complementary anatomical biomarkers for improving anatomical characterization and OSA risk stratification in this population.

## Supplementary Material

Supplementary Files

This is a list of supplementary files associated with this preprint. Click to download.

- SupplementaryMaterial.docx

## Figures and Tables

**Table 1.** Sample composition and OSA distribution. **A)** Composition of the euploid (EU) and Down syndrome (DS) groups included in the study. Age is reported as range and median [interquartile range, IQR]. **B)** Distribution of the DS subsample according to OSA severity. Age and apnea–hypopnea index (AHI) are reported as median [IQR] and median values, respectively. The number of males and females is indicated in brackets as (Males/Females).

		Table 1A. Sample composition			
Genetic Diagnosis		N (M/F)		Age, years range; Median [IQR]	
EU		63 (19/44)		18–84, 58.54 [51.74; 64.45]	
DS		101 (62/39)		18–62, 40.91 [31.09; 49.20]	
Total		164			
Table 1B. OSA distribution in DS					
OSA category		N (M/F)	Age Median [IQR]	AHI, Median	Total
No OSA (AHI < 5)		29 (14/15)	39.80 [29.75; 45.82]	1.6	29
OSA (AHI ≥ 5)	Mild OSA (AHI 5–29.9)	30 (18/12)	32.69 [30.00; 40.87]	10.65	62
	Severe OSA (AHI ≥ 30)	32 (24/8)	45.24 [39.90; 52.70]	48.15	
**Total**					91

## Data Availability

Raw magnetic resonance image data cannot be made available due to restrictions imposed by the ethics approval. Landmark data supporting the findings of this study are available from the corresponding authors upon reasonable request.

## Abbreviations

OSA: Obstructive sleep apnea
DS: Down syndrome
Aβ: Beta-amyloid
PSG: Polysomnography
EU: Euploid controls
AHI: Apnea-hypopnea index
MRI: Magnetic resonance imaging
ANOVA: Analysis of variance
GM: Geometric morphometrics
GPA: Generalized Procrustes Analysis
BMI: Body mass index
CVA: Canonical Variate Analysis
